# Focal Segmental Glomerulosclerosis Followed by Acute Hepatitis A Infection: Case Report

**DOI:** 10.3390/medicina59050819

**Published:** 2023-04-22

**Authors:** Min-Woo An, Jeong-Ju Yoo, Jin Kuk Kim, Ahrim Moon, Sang Gyune Kim, Young Seok Kim

**Affiliations:** 1 Division of Gastroenterology and Hepatology, Department of Internal Medicine, Soonchunhyang University Bucheon Hospital, Soonchunhyang University School of Medicine, Bucheon 14584, Republic of Korea; 2 Division of Nephrology, Department of Internal Medicine, Soonchunhyang University Bucheon Hospital, Soonchunhyang University School of Medicine, Bucheon 14584, Republic of Korea; 3 Department of Pathology, Soonchunhyang University Bucheon Hospital, Soonchunhyang University School of Medicine, Bucheon 14584, Republic of Korea

**Keywords:** hepatitis A virus, focal segmental glomerulosclerosis, extrahepatic, complication, proteinuria

## Abstract

*Background and Objectives*: Chronic viral hepatitis such as hepatitis B or hepatitis C is frequently related to nephropathies, yet acute hepatitis A virus (HAV) infection is an exception. *Materials and Methods*: A 43-year-old male presented with jaundice accompanied by nausea and vomiting. The patient was diagnosed with acute HAV infection. Although the liver function improved after conservative treatment, various symptoms such as proteinuria, hypoalbuminemia, generalized edema and pleural effusion persisted. Due to nephrotic syndrome, the patient was referred to the clinic of the nephrology department and a renal biopsy was performed. *Results*: The result of the renal biopsy was focal segmental glomerulosclerosis (FSGS) based on histology, electron microscopy and immunohistochemistry. Therefore, based on the clinical history and biopsy results, the patient was diagnosed as having FSGS aggravated by acute HAV infection. Proteinuria, hypoalbuminemia and generalized edema were improved after prednisolone treatment. *Conclusions*: Although less common, acute HAV infection can also present with an extrahepatic manifestation, for example, FSGS. Hence, clinical attention is required if proteinuria or hypoalbuminemia persists in patients with acute HAV infection.

## 1. Introduction

Viral hepatitis is associated with various types of extrahepatic manifestations [1]. These can occur in both acute and chronic liver diseases, and may precede or follow overt viral hepatitis [2]. However, extrahepatic manifestations of viral hepatitis are more frequently reported in chronic viral hepatitis forms such as hepatitis B virus (HBV) and hepatitis C virus (HCV) compared to hepatitis A virus (HAV) [3,4]. Specifically, there are numerous reports about glomerulonephritis following HBV or HCV infection. On the other hand, HAV is associated with acute hepatitis and does not progress to chronic hepatitis [5]. In addition, extrahepatic complications are relatively rare in HAV [1]. Although extrahepatic manifestations such as myocarditis and Guillain–Barre syndrome have been reported in acute HAV infections, there are very few reports of renal injury after HAV infection [6].

We report a case of a patient who presented with classical acute hepatitis A, whose liver profile quickly improved but who developed classical features consistent with nephrotic syndrome.

## 2. Case Presentation

A 43-year-old male visited a referral university hospital for jaundice and nausea. He was referred to an outpatient clinic of the gastroenterology department in a tertiary hospital due to jaundice and increased levels of liver enzymes detected during blood testing.

The patient’s medical history included dyslipidemia, fatty liver and hypothyroidism. He was taking levothyroxine, 0.05 mg daily for hypothyroidism. No medication was taken for dyslipidemia or fatty liver, and only lifestyle modification was recommended.

At the time of admission, he was 174 cm tall, weighed 74 kg and had a body mass index of 24.4 kg/m^2^. The blood pressure of the patient was slightly low, with a systolic blood pressure of 102 mmHg and a diastolic blood pressure of 65 mmHg. Mildly elevated body temperature (37.3 °C) and normal heart rate (78 beats per minute) with normal breathing rate (18 breaths per minute) was noted. The patient presented with nausea and jaundice, and epigastric discomfort. The patient had a soft abdomen on physical examination. Pitting edema was observed on both legs.

**Figure 1:**
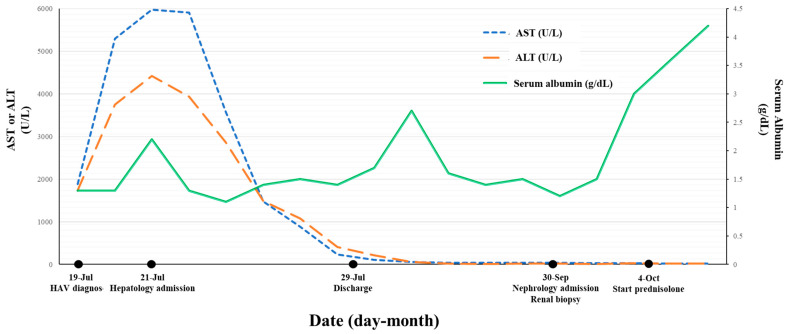
Clinical course of the patient.

A liver function test at baseline was markedly elevated with mild jaundice. Levels of aspartate aminotransferase (AST), alanine aminotransferase (ALT) and alkaline phosphatase (ALP) were 5971 U/L, 4419 U/L and 212 U/L, respectively. Prothrombin time-international normalized ratio (PT/INR) value was slightly increased to 1.62. The total bilirubin level was 2.14 mg/dL and the serum albumin level was decreased to 2.2 g/dL. Urinalysis showed proteinuria (3+) and microhematuria (3+). Spot urine total protein–creatinine ratio was 14,706. The trends of the patient’s blood tests are presented in Figure 1. The blood test results related to hepatitis virus are as follows: anti-HAV immunoglobulin M (IgM) (positive), anti-HAV immunoglobulin G (IgG) (negative), hepatitis B surface antigen (HBsAg) (negative), anti-HBs (positive), anti-HCV (negative), and anti-HEV IgM (positive). In addition, the test results for other diseases that can cause acute hepatitis other than viral hepatitis were as follows: Epstein–Barr virus (EBV) viral capsid antigen (VCA) IgM (negative), EBV VCA IgG (negative), EBV deoxyribonucleic acid (DNA) (negative), cytomegalovirus (CMV) IgM (negative), CMV IgG (negative), herpes simplex virus polymerase chain reaction (negative).

**Figure 2:**
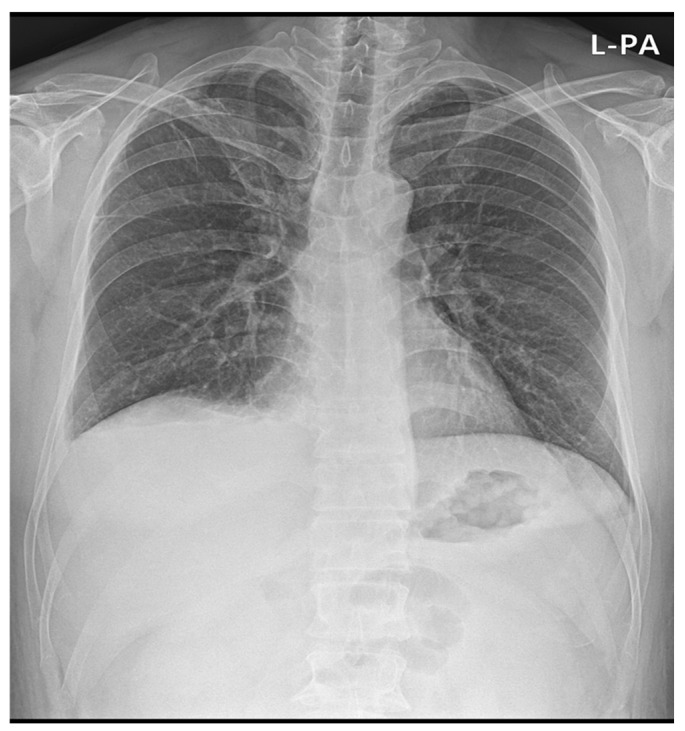
Chest X-ray of the patient showing pleural effusion.

In imaging examinations, the chest X-ray showed bilateral pleural effusion (Figure 2). Contrast-enhanced abdominal computed tomography (CT) revealed mild periportal edema and subserosal edema of the gallbladder with ascites, as well as bilateral pleural effusion. Abdominal ultrasound revealed decreased echogenicity and gallbladder wall thickening, probably related to acute hepatitis.

After 8 days of supportive treatment, gastrointestinal symptoms and jaundice caused by HAV improved, liver enzyme levels markedly decreased (AST 105 U/L, ALT 218 U/L, and ALP 201 U/L) and the patient was discharged. At the time of discharge, hypoalbuminemia (serum albumin 1.7 g/dL) and general edema had not improved.

**Figure 3:**
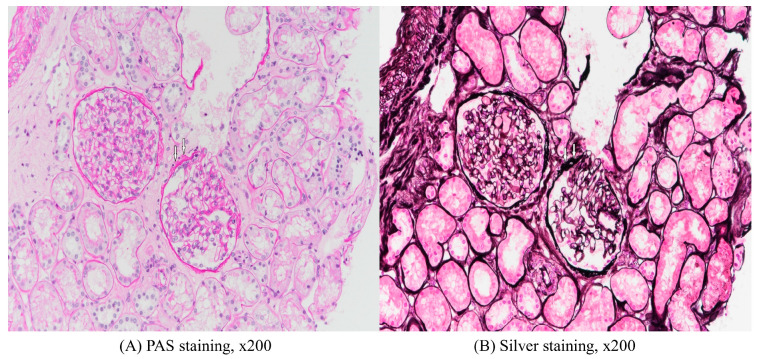
Results of renal biopsy, light microscopy (**A**) PAS staining, ×200, (**B**) Silver staining, ×200. Segmental glomerulosclerosis, lower right glomerulus (arrow). An adhesion is identified. The adhesion had a cell bridging between the laminated Bowman’s capsule and the glomerular basement membrane. It is suitable for the FSGS tip variant. The presence of Bowman capsule reaction helps distinguish a real adhesion from an artifact.

**Figure 4:**
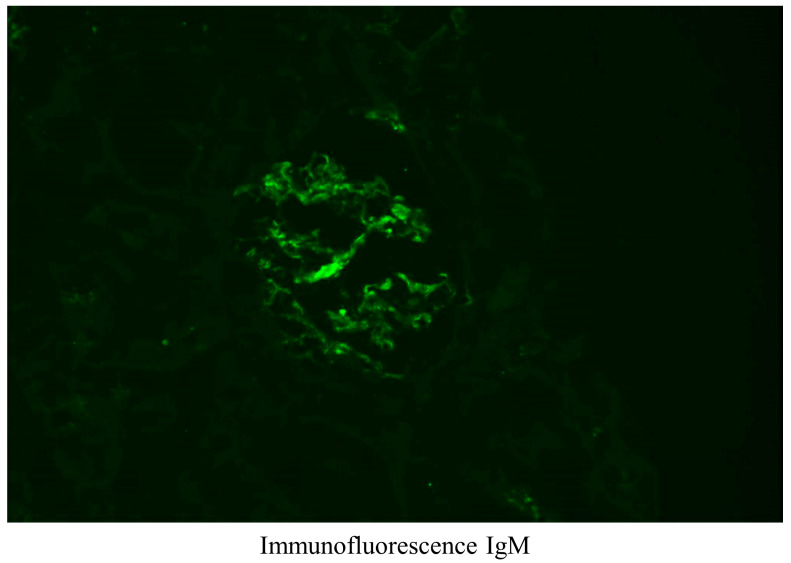
Results of renal biopsy, immunofluorescence IgM by immunofluorescence showed a mild positive (1+).

**Figure 5:**
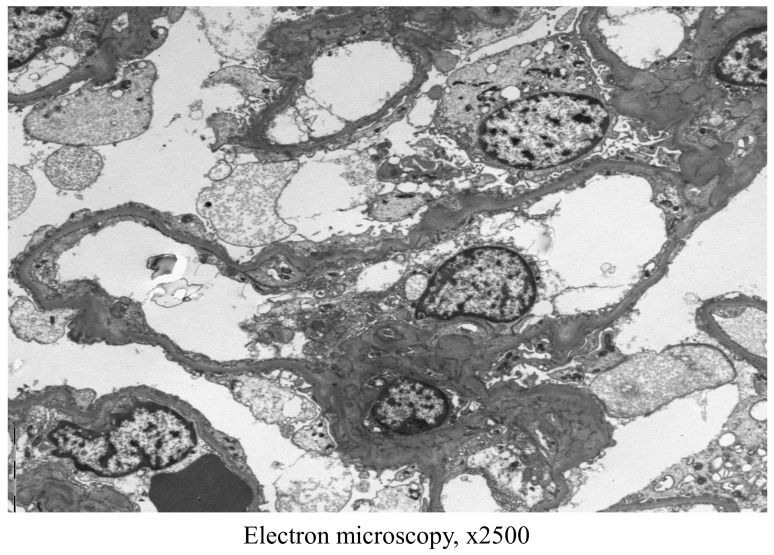
Results of renal biopsy, electron microscopy. Extensive foot process effacement accompanied by glomerular basement membrane wrinkling is seen.

After discharge, laboratory tests were performed at an outpatient clinic for 2 months at 2-week intervals, and proteinuria and hypoalbuminemia (serum albumin 1.5 g/dL) still did not improve. In addition, pleural effusion was continuously observed on chest X-ray. The result of a 24-h urinalysis was consistent with nephrotic syndrome (urine volume 2020 mL, creatinine clearance 139.8 mL/min, total protein 14,750 mg, creatinine 1610 mg, sodium 194 mmol). Protein electrophoresis showed hypogammaglobulinemia. As a result, 2 months after discharge from the hepatology department, the patient was re-admitted to the nephrology department and a kidney biopsy was performed. On light microscopy, 14 glomeruli were contained, 2 glomeruli showed global sclerosis and other glomeruli were slightly increased in size and normocellular. Mesangial matrix was not increased, while capillary loops were somewhat wrinkled. Tubules revealed mild atrophy with interstitial fibrosis and mild mononuclear infiltration. Arteries exhibited moderate arteriosclerosis with intimal thickening (Figure 3). Eight glomeruli processed for immunofluorescence analysis showed immunoglobulin (Ig) M (1+), complement component 1q (trace) and C4 (trace), and were negative for IgG, IgA, C3, kappa and lambda (Figure 4). On electron microscopy, glomerular basement membrane was moderately irregular in contour with moderate effacement of epithelial foot processes. Some equivocal electron dense deposits were found in a subendothelial lesion (Figure 5). Overall, renal biopsy was consistent with nephrotic syndrome due to focal segmental glomerulosclerosis (FSGS). Considering both the medical history and test results, the patient was finally diagnosed with acute hepatitis A-related FSGS.

The study protocol was approved by the Institutional Review Board of Soonchunhyang University Bucheon Hospital (SCHBC 2022-12-018, date of approval 10-January-2023), and conformed to the ethical guidelines of the World Medical Association Declaration of Helsinki. Written informed consent was obtained because of the retrospective design.

## 3. Results

The patient started to take prednisolone 60 mg daily for 8 weeks for empirical treatment of nephrotic syndrome. The dose of prednisolone was gradually reduced every 3 weeks. After the treatment, the patient’s albumin levels were maintained within the normal range (serum albumin 4.2 g/dL), and pitting edema was also improved along with proteinuria. Random urine urinalysis showed total protein–creatinine ratio < 200. Currently, the patient is being observed in the outpatient clinic.

## 4. Discussion

We are reporting a case of a patient with acute hepatitis A-induced FSGS, who developed proteinuria. Referring to the previous literature review, our case is the first in the world to report a FSGS type of glomerulonephritis caused by HAV.

In chronic hepatitis B or C infection, glomerulonephritis is one of the most common extrahepatic manifestations. In particular, it has been reported that chronic hepatitis C has a higher rate of proteinuria found in urinalysis than the normal population, but the frequency of clinically serious renal disease is quite rare [7,8]. Acute tubular necrosis and acute tubulointerstitial nephritis were reported in most of the cases, and glomerular diseases such as immune complex mesangial proliferative glomerulonephritis or IgA nephropathy were rarely reported. HAV infection usually resolves spontaneously without specific treatment, but extrahepatic manifestations may occur in rare cases. Theoretically, renal injury may also occur in HAV, but much less frequently than in other types of viral hepatitis. Previous cases of renal injury caused by HAV are summarized in Table 1 [9,10,11,12,13,14,15,16,17,18,19]. IgA nephropathy was the most common renal injury caused by HAV and minimal change disease, as well as mesangial proliferation were also very common, while FSGS like our case has not been reported.

Viral infections can cause FSGS, and podocyte injury is known to be the main mechanism of FSGS [24,25]. Viral infections that can cause FSGS include human immunodeficiency virus (HIV), Parvovirus B19, HBV and HCV. In HIV, the main pathophysiology of FSGS is the expression of HIV transgenes in podocytes, dedifferentiation and apoptosis of podocytes caused by apolipoprotein L1 variant transcription and accumulation of immune complex in glomerular capillaries [26,27]. Parvovirus B19-associated FSGS exhibits a loss of differentiation and a gain of proliferation in podocytes [28]. In HBV-associated FSGS, the virus may have a direct pathogenic effect, as HBV-DNA was found in urinary podocytes using real-time polymerase chain reaction [29]. The mechanism of HCV-associated FSGS is not clear, but it may be caused by direct podocyte injury similar to HIV [29].

On the other hand, unlike other viruses, the mechanism by which HAV causes kidney injury is unclear. Hepatitis A antigen was found in renal glomeruli in marmosets, but has not yet been found in humans [30]. HAV infects using T cell immunoglobulin mucin domain 1 (TIM1), known as kidney injury molecule 1 (KIM1), which can be highly expressed by renal cells. KIM1 is a type I transmembrane protein with an immunoglobulin and mucin domain whose expression is markedly upregulated in the proximal tubule cells [31,32]. However, HAV has not been detected in renal tissue [10]. Thus, further research is needed to understand why HAV caused direct podocyte injury, as in our case.

Since most cases of HAV recover without complications and extrahepatic manifestations are very rare, clinicians tend not to actively evaluate organs other than the liver. Especially, hepatitis A rarely manifests with nephrotic syndrome [33]. However, since nephrotic syndrome does occur in some cases such as ours, clinicians should pay attention. The clinical definition of adult nephrotic syndrome includes massive proteinuria (≥3.5 g/day), hypoalbuminemia (serum albumin ≤ 3.0 g/dL), edema and dyslipidemia (hyper-LDL cholesterol). The predominant symptom of nephrotic syndrome is edema, and also, in our case, pretibial edema was observed from the time of admission. Looking at the existing cases of HAV-induced renal injury (Table 1), proteinuria was present in eight out of nine cases (88.9%) including our case. In laboratory findings, hematological abnormalities such as hypoalbuminemia, hypercholesterolemia, renal and liver dysfunction, electrolyte disorders, coagulation/fibrinolysis disorders, hormonal disorders and anemia are usually found in patients with nephrotic syndrome [34]. Clinically, nephrotic syndrome should be suspected when the above symptoms are present, especially proteinuria and edema. Renal biopsy is usually considered useful and recommended for definitive diagnosis. In fact, renal biopsy was performed in all nine existing cases (Table 1). Table 1 shows that acute hepatitis A cases reported in Korea are concentrated in a specific age group (27–43 years). Regarding the high incidence of acute HAV in a specific age group, the Korean government has included the hepatitis A vaccine in the national mandatory vaccination program for newborns born after January 2012. Until then, it was not a mandatory vaccine. Therefore, in Korea, the current rate of antibody positivity for hepatitis A is low among people in their 20s and 40s. In 2015, the antibody positivity rate for Koreans in their 20s and 30s was only 12.6% and 31.8%, respectively. Because of this phenomenon, it seems that the ages of HAV patients in Korea are concentrated between 27–43 years old.

In our case report, it’s important to note that the patient had low levels of albumin and pleural effusion from the start of their hospitalization. It is known that hypoalbuminemia can lead to pleural effusion, so when doctors notice effusions in patients with low albumin levels, they should investigate other possible causes with thorough clinical evaluations [35,36]. Although the rate of pleural effusion may vary depending on the underlying condition or disease that caused the hypoalbuminemia, it is estimated that up to 90% of patients with nephrotic syndrome have hypoalbuminemia [37,38]. Thus, if a patient with acute hepatitis rapidly improves, but hypalbuminemia persists and edema develops, causes of renal protein loss and renal disease should be incorporated in the differential diagnosis.

Treatment should be individualized for each patient. In six out of nine cases (66.7%), renal injury was improved only with supportive treatment, which seems to be related to the pathology of renal injury. Most cases of IgA nephropathy, minimal change disease and mesangial proliferation have a good prognosis. However, in the case of interstitial nephritis or FSGS as in this case, active treatment such as steroid treatment and dialysis is required. Regarding our case, about half of the cases of FSGS are non-responders to steroid treatment. The responsive rate and renal prognosis vary across the variant types of FSGS. In the tip variant, steroid response was present in 97% and the remission rate was 75%, being the highest among FSGS and the progression rate to end-stage renal disease was 5.7% [33]. For the treatment of secondary FSGS, the priority is to treat the underlying disease [39,40,41]. However, if nephrotic syndrome persists even after the causative disease has improved, as in the idiopathic nephrotic syndrome treatment, steroid therapy should be considered first, followed by immunosuppressive therapy according to steroid dependence/resistance [34].

## 5. Conclusions

In conclusion, although not common, renal injury may occur as one of the complications of HAV infection. Therefore, hepatitis A patients with edema, proteinuria and hypoalbuminemia require special clinical attention. Further research on the mechanism of why HAV causes such FSGS is needed.

## Figures and Tables

**Table 1:** Characteristics of HAV-associated glomerular diseases based on prior case reports.

Case Report	Demographics			Course of Illness				Initial Values		
	Location	Sex	Age (yr)	Symptom	Biopsy	Treatment	Outcome	AST/ALT (U/L)	Albumin (g/dL)	Proteinuria
Our case	Rep. of Korea	M	43	Jaundice, Nausea	Focal segmental glomerulosclerosis	Prednisolone	Improved	5971/4419	2.2	Presence
Han et al., 2010 [12]	Rep. of Korea	F	30	General weakness	IgA nephropathy	Losartan	Improved	1917/2944	1.1	Presence
Hong et al., 2010 [13]	Rep. of Korea	F	30	General weakness	IgA nephropathy	Supportive	Improved	1917/2944	1.1	Presence
Lee et al., 2004 [20]	Rep. of Korea	M	27	Anorexia	IgA nephropathy	Supportive	Improved	388/1566	Normal	Trace
Han et al., 2007 [21]	Rep. of Korea	F	31	Fever, myalgia	IgA nephropathy and Interstitial nephritis	Hemodialysis	Improved	17,215/6874	Normal	Presence
Kron et al., 1984 [22]	USA	M	5	Asymptomatic proteinuria	Minimal change disease	Supportive	Improved	70/NA	Normal	Presence
Pal et al., 2011 [19]	India	M	3	Jaundice	Mesangial proliferation	Supportive	Improved	4695/3836	Normal	Presence
Demircin et al., 1998 [23]	Turkey	M	7	Jaundice, dyspnea	Mesangial proliferation	Supportive	Improved	65/62	Normal	Presence
Demircin et al., 1998 [23]	Turkey	M	6	Jaundice, dyspnea	Mesangial proliferation	Supportive	Improved	70/86	Normal	Presence

Abbreviations: HAV, hepatitis A virus; yr, year; NA, non-available.

## Data Availability

The datasets used and/or analyzed during the current study are available from the corresponding author on reasonable request.
